# Effects of TGF-β1 and IL-1β on expression of ADAMTS enzymes and TIMP-3 in human intervertebral disc degeneration

**DOI:** 10.3892/etm.2013.1348

**Published:** 2013-10-15

**Authors:** SHI-LONG WANG, YONG-LIN YU, CHAO-LIANG TANG, FEI-ZHOU LV

**Affiliations:** Department of Orthopedics, Huashan Hospital Affiliated to Fudan University, Shanghai 200040, P.R. China

**Keywords:** nucleus pulposus, transforming growth factor-β1, interleukin-1β, tissue inhibitor of metalloproteinase 3, a disintegrin and metalloproteinase with thrombospondin motifs

## Abstract

The aim of this study was to investigate the effects of transforming growth factor-β1 (TGF-β1) and interleukin-1β (IL-1β) on the expression of a disintegrin and metalloproteinase with thrombospondin motifs (ADAMTS) enzymes and their inhibitor, tissue inhibitor of metalloproteinase 3 (TIMP-3), in human intervertebral disc (IVD) degeneration. Cells from patients with IVD degeneration were cultured with Dulbecco’s modified Eagle’s medium with Ham’s F12 nutrient mixture (DMEM/F12) medium at 37°C in a 5% CO_2_ incubator. Cell proliferation was measured by cell counting kit-8 assays with varying concentrations of TGF-β1 and IL-1β in a time-response experiment. The mRNA and protein expression levels of ADAMTS-4, ADAMTS-5 and TIMP-3 were detected with qPCR and western blot analysis, respectively. The present study demonstrated that TGF-β1 promoted nucleus pulposus (NP) cell proliferation, decreased the expression of ADAMTS-4 and -5 and increased the expression of TIMP-3. By contrast, the IL-1β treatment inhibited NP cell proliferation and significantly increased the expression of ADAMTS-4 and -5. However, IL-1β appeared to have no marked effect on the expression of TIMP-3. This study suggests that TGF-β1 and IL-1β are involved in the synthesis and degradation of the extracellular matrix and may act as potential therapeutic targets for the prevention or reversal of IVD degeneration.

## Introduction

The human intervertebral disc (IVD) is an important component of the spinal column and its dysfunction leads to lower back pain that may reduce the patient’s quality of life (1). The degeneration of the IVD is a complex process characterized by a series of biochemical and structural changes in the nucleus pulposus (NP) (2). IVD degeneration often leads to the unclear boundary between the annulus fibrosus and NP, composition changes of the collagen fibers, as well as a reduction in the proteoglycan content and loss of water (3). NP cells are chondrocyte-like cells and secrete a complex extracellular matrix (ECM) that predominantly consists of proteoglycan and fibrillar collagen. Aggrecan, one type of proteoglycan, maintains the normal structure, metabolism and biomechanical function of the disc (4). Glycosaminoglycan (GAG), a component of proteoglycans, contains large quantities of water, which allows the NP to be flexible enough to withstand loading. The considerable loss of GAG in the process of disc degeneration results in reductions in water content and NP elasticity, which lead to the dysfunction of IVD biomechanics (5). A previous study revealed that the loss of GAG predominantly results from the increased hydrolysis of proteoglycans (6). Aggrecan synthesis and degradation are in a dynamic equilibrium that maintains the physiological function of the IVD. However, this dynamic balance gradually becomes distorted under the influence of age and stress, resulting in the degeneration of human IVDs. A previous study has demonstrated that the expression and activity of matrix-degrading enzymes are increased and elicit degradation of the ECM in the degenerative disc (7).

There are two predominant degrading enzymes that are able to hydrolyze aggrecan core proteins; these are matrix metalloproteinases (MMPs) and aggrecanases (8). MMPs have been demonstrated to hydrolyze aggrecan and collagen, as well as fibronectin proteins. Aggrecanase belongs to the a disintegrin and metalloproteinase with thrombospondin motifs (ADAMTS) family and has the ability to degrade aggrecan. In the NP matrix, aggrecan is predominantly degraded by the ADAMTS family members ADAMTS-4 and ADAMTS-5 (8,9). Tissue inhibitor of metalloproteinase 3 (TIMP-3), inhibits aggrecanase and therefore, an increase in the level of TIMP-3 slows disc degeneration. Studies have demonstrated that growth factors, including transforming growth factor-β1 (TGF-β1), and cytokines, such as interleukin-1β (IL-1β), are involved in ECM metabolism and cell proliferation (10,11). The present study investigated the effects of TGF-β1 and IL-1β on the expression levels of ADAMTS enzymes and their inhibitor TIMP-3 in human IVD degeneration, and aimed to identify a potential therapeutic target for human IVD degeneration.

## Materials and methods

### Cell isolation and culture

This study was approved by the ethics review board of Fudan University, affiliated to Huashan Hospital (Shanghai, China). All patients provided consent for involvement in this study. IVD specimens were obtained from the Spinal Surgery Center of Huashan Hospital. The degenerated IVD was classified by Christian MRI standard (12)and samples were obtained from six donors (three females and three males; average age, 34.4 years). Specimens were transported in a sterile tube to the laboratory <30 min after surgical removal. The annulus fibrosus and transition zone were removed using a scalpel. The NP tissue was carefully separated from the upper and lower vertebral cartilage under a binocular microscope (Olympus SZH, Tokyo, Japan). NP tissue was rinsed three times in D-Hank’s solution to remove residual debris. The tissue was then cut into 1-mm^3^ sections using a scalpel. The sample was then digested for 45 min at 37°C in 0.25% tryptase, followed by 4 h in 0.2% collagenase Type II. The digested sample was then filtered through a 75-μm cell-strainer and cultured in T25 flasks at a density of 1×10^4^ cells/ml in Dulbecco’s modified Eagle’s medium with Ham’s F12 nutrient mixture (DMEM/F12), containing 10% fetal bovine serum (FBS; HyClone, Rockford, IL, USA) in an incubator at 37°C with 5% CO_2_. The medium was changed every 72 h. When cultures demonstrated an 80% confluency, cells were trypsinized and a split ratio of 1:2 was used for subculturing.

### Determination of cell proliferation following treatment with TGF-β1 and IL-1β

The NP cell proliferation was analyzed using a cell containing kit-8 (CCK-8) assay (Sigma, St. Louis, MO, USA). The CCK-8 assay is based on the reduction of a water-soluble tetrazolium salt (WST-8) into a yellow soluble formazan product by metabolically active cells. The colored product accumulates in the culture medium rather than in the cells, allowing the assessment of cell growth at various times of culture. Upon reaching 80% confluency, the cells (passage 3) were trypsinized and cultured in 96-well plates containing 0.1 ml DMEM/F12 with 10% FBS, per well. The control group was cultured in DMEM/F12 containing 10% FBS, while the treatment group was cultured in DMEM/F12 containing 10% FBS and treated with IL-1β or TGF-β1 at varying concentrations (0.1, 1 and 10 ng/ml) for varying times (4, 8, 16, 24 and 48 h). The absorbance values of the culture medium of the samples were recorded at 450 nm, using a microplate reader (FluoDia T70; Photon Technology International, Lawrenceville, NJ, USA).

### RNA extraction and qPCR

Total RNA, of the control and treatment groups, was isolated with the TRIzol^®^ Reagent kit (Invitrogen Life Technologies, Carlsbad, CA, USA). cDNA was synthesized with reverse transcription PCR using: 2 μg RNA, 1 μl 0.5 μg Oligo (dT) 15 Primer (Promega Corporation, Madison, WI, USA), 1 μl of 10 mM dNTP mix, 5 μl of M-MLV 5X reaction buffer and 1 μl of 200 U M-MLV reverse transcriptase in RNase free water (final volume, 25 μl). All reactions were prepared according to the manufacturer’s instructions and were incubated at 42°C for 60 min.

qPCR was performed for ADAMTS-4, ADAMTS-5, TIMP-3 and glyceraldehyde 3-phosphate dehydrogenase (GAPDH) using the SYBR^®^-Green PCR Master mix kit (Applied Biosystems Inc., Foster City, CA, USA) in an ABI 7300 sequence detection system (Applied Biosystems). All the primers (Table I) were designed with the Primer Premier 5.0 software (Premier Biosoft, Palo Alto, CA, USA) according to the cDNA sequences published in GenBank.

### Western blot analysis

The expression levels of ADAMTS-4, ADAMTS-5 and TIMP-3 were detected by western blot analysis. Briefly, the human NP cells from the control and treatment groups were harvested after 48 h of treatment with TGF-β1 and IL-1β, and the protein concentration was determined using a Bicinchoninic Acid Protein assay kit (Pierce Biotechnology Inc., Rockford, IL, USA). Total protein (30 μg) was denatured at 100°C for 5 min and loaded into each well of a 10% sodium dodecyl sulfate-polyacrylamide gel. Following electrophoresis, the protein was transferred onto a nitrocellulose membrane using a charge of 250 mA for 60 min. The membrane was blocked in 5% milk in Tris-buffered saline (TBS) for 1 h at room temperature. The primary antibodies including ADAMTS-4/5 (Abcam, Cambridge, UK) and TIMP-3 (Santa Cruz Biotechnology, Inc., Santa Cruz, CA, USA) were added and incubated with the membrane overnight at room temperature, and then washed three times with TBS and Tween 20 (TBST). The anti-rabbit secondary antibody (Santa Cruz Biotechnology, Inc.) was added to the membrane, which was then incubated for 2 h at room temperature with gentle agitation. The membrane was washed three times with TBST for 10 min per wash. The bands were visualized using an enhanced chemiluminescence kit (Amersham, Arlington Heights, IL, USA) following exposure to an X-ray film.

### Statistical analysis

Data are presented as the mean ± SEM and were analyzed using SPSS software (SPSS, Inc., Chicago, IL, USA). Student’s t-test and analysis of variance were used for comparisons between groups. P<0.05 was considered to indicate a statistically significant difference.

## Results

### Effects of TGF-β1 and IL-1β on the proliferation of human NP cells

**Figure 1:**
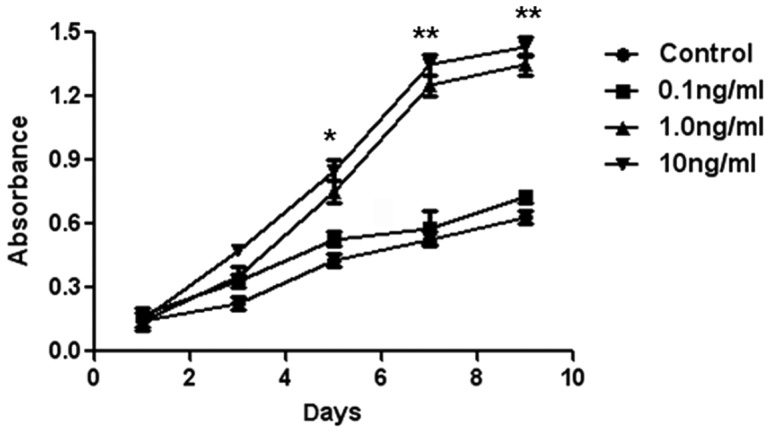
Effects of TGF-β1 on the proliferation of human NP cells. Treatment of human NP cells with different concentrations of TGF-β1 for nine days. The concentrations of TGF-β1 were 0.1, 1.0 and 10.0 ng/ml. Absorbance values were measured at 450 nm using a spectrophotometer and the results shown are from three independent experimental cultures. ^*^P<0.05 and ^**^P<0.01 compared with control. TGF-β1, transforming growth factor-β1; NP, nucleus polposus.

**Figure 2:**
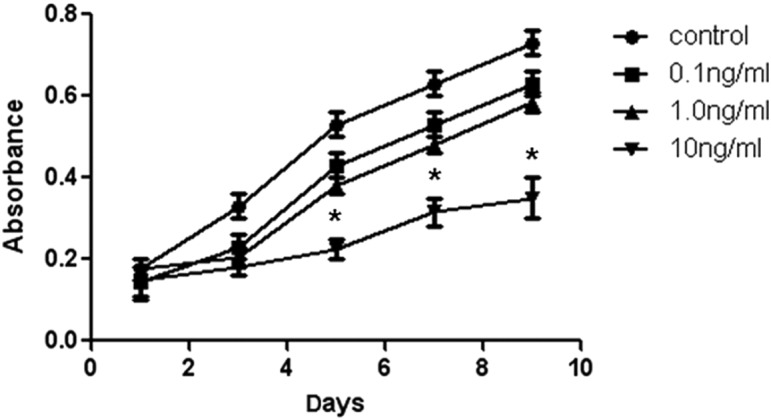
Effects of IL-1β on the proliferation of human NP cells. Treatment of human NP cells with different concentrations (0.1, 1.0 and 10.0 ng/ml) of IL-1β for nine days. Absorbance values were measured at 450 nm using a spectrophotometer and the results shown are from three independent experimental cultures. ^*^P<0.05 and ^**^P<0.01 compared with control. IL-1β, interleukin-1β; NP, nucleus polposus.

To determine the effect of TGF-β1 and IL-1β on NP cell proliferation, a time-response experiment of human NP cell proliferation was performed using the CCK-8 assay in 96-well plates. Fig. 1 demonstrates that treatment with TGF-β1 at a concentration of 0.1 ng/ml exhibited no significant effect on the proliferation of NP cells. However, a high concentration of TGF-β1 (1 and 10 ng/ml) treatment significantly promoted cell proliferation. Furthermore, Fig. 2 shows that 10 ng/ml IL-1β significantly inhibited the human NP cell proliferation, while lower concentrations of IL-1β did not exhibit a marked effect. These results revealed that 1 or 10 ng/ml for TGF-β1 and 10 ng/ml for IL-1β appeared to be the optimal concentrations for the inhibition of human NP cell proliferation and were applied in the subsequent experiment.

### Effects of TGF-β1 on the expression of ADAMTS enzymes and TIMP-3 in human NP cells

**Figure 3:**
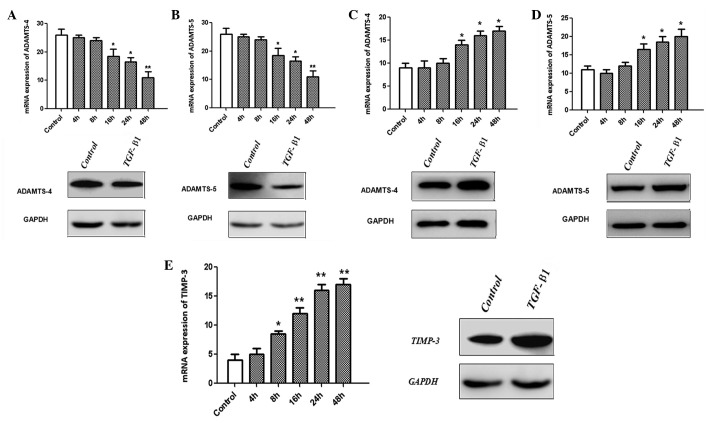
Effects of TGF-β1 on the expression of ADAMTS enzymes and TIMP-3 in human nucleus pulposus cells evaluated at the mRNA and protein expression levels by real time PCR and western blotting, respectively. Expression levels of ADAMTS-4 and ADAMTS-5 following treatment with 1.0 ng/ml TGF-β1 (A and B, respectively) and 10.0 ng/ml TGF-β1 (C and D, respectively). mRNA and protein expression levels of TIMP-3 following treatment with 1.0 ng/ml TGF-β1 (E). ^*^P<0.05 and ^**^P<0.01, compared with control. TGF-β1, transforming growth factor-β1; ADAMTS, a disintegrin and metalloproteinase with thrombospondin motifs; TIMP-3, tissue inhibitor of metalloproteinase 3; GAPDH, glyceraldehyde 3-phosphate dehydrogenase.

Human IVD degeneration is accompanied by the increased expression of degrading enzymes, including ADAMTS. Therefore, the effect of TGF-β1 on the expression of ADAMTS-4 and ADAMTS-5, the enzymes that are predominantly involved in the matrix degrading process, was examined. qPCR showed that the expression of ADAMTS-4 and -5 mRNA significantly decreased in a time-dependent manner following treatment with TGF-β1 at a concentration of 1 ng/ml. Western blot analysis also demonstrated that the protein expression of ADAMTS-4 and -5 significantly decreased following treatment with TGF-β1 for 48 h (Fig. 3A and B). However, treatment with 10 ng/ml TGF-β1 appeared to increase the expression of the ADAMTS enzymes (Fig. 3C and D), indicating that a high concentration of TGF-β1 may produce an adverse effect. Subsequently, the expression of the MMP inhibitor, TIMP-3, was examined and the results revealed that TGF-β1 treatment at concentrations of 1 and 10 ng/ml (data not shown for the latter) significantly increased the expression of TIMP-3 at the mRNA level in a time-dependent manner. The western blot analysis revealed that the TIMP-3 protein levels were increased following treatment with 1 ng/ml TGF-β1 for 48 h (Fig. 3E).

### Effects of IL-1β on the expression of ADAMTS enzymes and TIMP-3 in human NP cells

**Figure 4:**
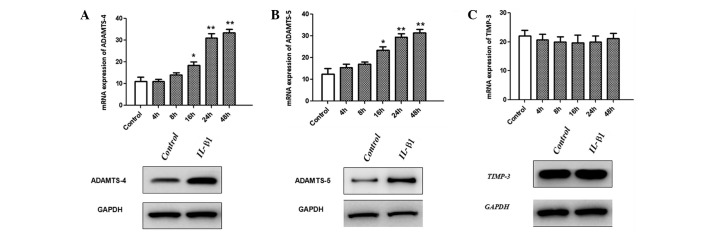
Effects of IL-1β on the expression of ADAMTS enzymes and TIMP-3 in human nucleus pulposus cells. The mRNA levels and protein expression was measured by qPCR and western blot analysis for (A) ADAMTS-4, (B) ADAMTS-5 and (C) TIMP-3 with IL-1β treatment at a concentration of 10.0 ng/ml. ^*^P<0.05 and ^**^P<0.01 compared with control. IL-1β, interleukin-1β; ADAMTS, a disintegrin and metalloproteinase with thrombospondin motifs; TIMP-3, tissue inhibitor of metalloproteinase 3; GAPDH, glyceraldehyde 3-phosphate dehydrogenase.

The effects of IL-1β on the expression of ADAMTS-4, ADAMTS-5 and TIMP-3 in the NP cells were examined. qPCR revealed that the expression of ADAMTS-4 and -5 significantly increased in a time-dependent manner following treatment with IL-1β at a concentration of 10 ng/ml. Western blot analysis also demonstrated that the protein expression levels of ADAMTS-4 and -5 significantly increased with 48 h of IL-1β treatment (Fig. 4A and B). However, IL-1β appeared to have no marked effect on the expression of TIMP-3 (Fig. 4C).

## Discussion

IVD degeneration is characterized by morphological changes and is closely associated with the degradation of the ECM, which leads to a reduction in the NP cell population (13). The proteoglycan (predominantly aggrecan) content is important in maintaining proper IVD function, particularly in the NP (5). The role of MMPs in the articular cartilage and IVD ECM has been studied for a number of years and is relatively well understood. Several members of the aggrecanase enzyme family that participate in ECM breakdown have been identified, and studies have shown that aggrecanases may be involved in the initiation and progression of IVD degeneration (14). ADAMTS-4 and ADAMTS-5 are critical in the breakdown of ECM in the degenerative disc (15,16). The expression of TIMP-3 has been identified to be reduced in degenerated NP and annulus fibrosus samples and is known to inhibit the two ADAMTS enzymes and numerous other matrix proteinases, such as MMPs (17).

Cytokines are critical in ECM synthesis as they regulate cell metabolism. TGF-β1 has been shown to stimulate NP cell proliferation and has been applied in tissue engineering for the repair of IVD. TGF-β1 treatment has been demonstrated to increase mitogen-activated protein kinase activity and sex determining region Y-box 9, aggrecan, and collagen type II gene expression (18). In addition, another study reported that in IVD cells, stimulation by TGF-β1, epidermal growth factor and insulin-like growth factor may significantly increase proteoglycan synthesis and promote cell proliferation (17,19). IL-1β plays a biological role by promoting cell metabolism and inhibiting anabolism. It stimulates cells to produce a variety of neutral proteases, particularly MMPs, and reduce the expression of proteoglycan in the ECM (11,13,20). Studies have shown that IL-1β is expressed in degenerating discs and is involved in the catabolism of the ECM. Tsuji *et al*(21) showed that the stimulation of NP cells with IL-1β for 24 h significantly increased the expression level of ADAMTS-4 mRNA and demonstrated that IL-1β is involved in the degradation of ECM in the IVD cells. Therefore, it was hypothesized that the inhibition of IL-1β expression may be a treatment option for IVD degeneration. The present study aimed to investigate the involvement of TGF-β1 and IL-1β in the proliferation of NP cells and expression of genes involved in ECM synthesis. The results demonstrated that TGF-β1 promoted NP cell proliferation. Furthermore, the downregulation of ADAMTS-4 and -5 and upregulation of TIMP-3 was demonstrated following treatment with 1 ng/ml TGF-β1. However, treatment with a higher concentration of TGF-β1 (10 ng/ml) appeared to increase the expression of ADAMTS enzymes, indicating that an over-dose of TGF-β1 treatment may produce an adverse effect. Conversely, IL-1β treatment inhibited NP cell proliferation and significantly increased the expression of ADAMTS-4 and -5. However, IL-1β appeared to have no marked effect on the expression of TIMP-3.

In conclusion, this study demonstrated that the cytokines TGF-β1 and IL-β, are involved in the synthesis and degradation of the ECM and may be potential therapeutic targets for the prevention or reversal of IVD degeneration.

## Figures and Tables

**Table I:** Primers used in qPCR experiments.

Gene	Forward primer sequence (5′-3′)	Reverse primer sequence (5′-3′)	Product size (bp)
GAPDH	AGAAGGCTGGGGCTCATTTG	AGGGGCCATCCACAGTCTTC	258
TIMP-3	AGATTCTGACTTCTTCCTCCG	TGGGCTAGATTTCCAGCAAGTG	215
ADAMTS-4	ACCCAAGCATCCGCAATC	CAGGTCCTGACGGGTAAACA	200
ADAMTS-5	GCAGTATGACAAGTGCGGAGT	CAGGGCTAAATAGGCAGTGAA	161

GAPDH, glyceraldehyde 3-phosphate dehydrogenase; TIMP-3, tissue inhibitor of metalloproteinase 3; ADAMTS, a disintegrin and metalloproteinase with thrombospondin motifs.
